# Dietary characterization of terrestrial mammals

**DOI:** 10.1098/rspb.2014.1173

**Published:** 2014-08-22

**Authors:** Silvia Pineda-Munoz, John Alroy

**Affiliations:** Department of Biological Sciences, Macquarie University, Sydney, New South Wales 2109, Australia

**Keywords:** mammal ecology, mammal palaeoecology, dietary specialization, ecomorphology

## Abstract

Understanding the feeding behaviour of the species that make up any ecosystem is essential for designing further research. Mammals have been studied intensively, but the criteria used for classifying their diets are far from being standardized. We built a database summarizing the dietary preferences of terrestrial mammals using published data regarding their stomach contents. We performed multivariate analyses in order to set up a standardized classification scheme. Ideally, food consumption percentages should be used instead of qualitative classifications. However, when highly detailed information is not available we propose classifying animals based on their main feeding resources. They should be classified as generalists when none of the feeding resources constitute over 50% of the diet. The term ‘omnivore’ should be avoided because it does not communicate all the complexity inherent to food choice. Moreover, the so-called omnivore diets actually involve several distinctive adaptations. Our dataset shows that terrestrial mammals are generally highly specialized and that some degree of food mixing may even be required for most species.

## 1. Introduction

Reconstructing the nutritional requirements of an ecosystem's components is essential for understanding its function and for designing further biological studies. Accurate information about feeding behaviour is also a pre-requisite for research on ecomorphology; making inferences about the fossil record [1–3]; inferring the climate and ecological context of fossil localities, which enables tracking global climate change throughout the geological time scale [4–6]; and modelling food webs [7–10].

Contemporary mammals are extraordinarily diverse, having adapted to fill almost all available ecological niches [3,11,12]. They also play a key role in the dynamics of the ecosystems in which they live [13,14]. Therefore, it is crucial to reconstruct the trophic relationships between mammals and the other components of their ecological communities [15]. Many researchers have attempted to analyse the morphology, ecology and palaeoecology of mammals based on their dietary preferences [4,16,17]. Some have used basic feeding classifications equating with classic trophic levels—herbivores, carnivores and omnivores, plus a few variations [2,3,16,18]. Others distinguished multiple food resources within diets [1,17,19]. However, none of the previous workers performed multivariate analyses to support their dietary categorizations. This situation hinders comparing results from different ecomorphological studies.

Here, we offer a new classification scheme of the feeding preferences of modern mammals. This scheme is intended to provide a standardized foundation for research concerning ecomorphology and the functioning of global terrestrial ecosystems.

## 2. Material and methods

We built a database summarizing the dietary preferences of terrestrial mammals using published data. Data were compiled from primary resources (see the electronic supplementary material, S1) that were identified using academic search engines and databases. Quantitative information on diet can be generated using any of three major sampling methodologies: (i) time spent eating each food resource, (ii) faeces composition, and (iii) stomach contents. The analysis of time budgets is far from replicable as it is subject to observer error and personal biases [20,21]. Moreover, food resources have variable processing and/or acquisition times that are not necessarily proportional to the volume of food consumed, and it is impossible to sample some mammal species in this way [21]. Faeces contents are easier to evaluate because less time is required and there is no direct interaction with the animals [22,23]. However, different kinds of food have different responses to digestive processes and these responses are highly variable among species and individuals. Thus, these problems may cause important sampling biases [23,24]. Analysis of stomach/gut contents usually demands sacrificing a large number of animals, which makes it difficult to work with threatened species. Thus, fewer studies use this sampling methodology [22–24]. However, this method provides direct information, with all ingested food potentially being found and degradation from digestive processes being minimal [22,24].

We define ‘diet’ as the average variety of food ingested over the entire lifetime of the individuals of a species. However, data of this exact kind do not exist because observations of feeding behaviour over short intervals, of scats or of gut contents only apply to short-term diets of a few individuals in one or a few places. We believe this problem is not insurmountable because relatively minor variation is seen within particular datasets and because we have taken steps in order to compile a dataset as close to our dietary definition as possible.

Because we were able to retrieve a significant amount of data of volumetric percentages of stomach contents, we restricted our analysis to data of this nature. Unfortunately, some of the references did not report the sample size of the analysed species. We nonetheless included all collected data in our analysis because we felt that sample sizes were likely to be reasonable regardless of whether they were reported. Additionally, we used gross averages for each of the species independently of the season and locality in order to approximate the average diet for the species. Literature reported the average across all seasons for 16% of the species in the dataset; seasonal breakdowns were available for 31% of the data records, and in these cases we calculated the average across all seasons; 53% of the species lacked any data regarding seasonal changes in diet, which meant that only a single record could be employed. There was information for 8% of the species regarding multiple localities, and in those cases we similarly used the average across all the localities. We ultimately found data on percentage stomach content volume for 139 mammalian species (electronic supplementary material, S1).

We used the taxonomic classification of Wilson & Reeder [12] to standardize the nomenclature. Classifying food resources was a complex task. Some authors reported the taxonomic attribution of the consumed items within the stomach contents, while others classified them within broad categories. Moreover, resource classifications varied significantly among authors. For example, some distinguished between grass, forbs, leaves and branches, while others included all of the above in a single food category (vegetation). Previous literature focused on classifying herbivore diets in order to infer browsing and grazing behaviours [25]. However, this kind of information was generally not available in the stomach content datasets we recovered. Because we were focusing on the diet of whole terrestrial mammalian faunas and wanted to maximize the extent of our survey, we therefore felt comfortable with not breaking down herbivores into browsers and grazers.

We established eight feeding resource groups easily recognizable in all terrestrial ecosystems: seeds, invertebrates, vertebrates, fungus, flowers and gum, roots and tubers, green plants and fruit (electronic supplementary material, S2 gives details). We excluded values pertaining to miscellaneous undefined food resources to make the data comparable among species.

We used the R environment [26] for statistical analysis and construction of tables and figures. We carried out principal components analysis (PCA) to identify the variables that best summarize the different dietary specializations. Factor analysis was also applied but is not discussed further because it provided similar results. Unfortunately, raw percentage values are non-independent because they must add up to 100, which make them computationally unsuitable for PCA. More importantly, PCA tends to underweight variables if the percentages are consistently low because the lower bound of zero compresses potential variance. This property masks the contribution of rare dietary preferences. We solved the problems of non-independence and underweighting by rescaling each variable as a *z*-score before carrying out the PCA. We then undertook unweighted pair group method with arithmetic mean (UPGMA) cluster analysis based on Euclidean distances among the PCA scores. Compared to more arbitrary manipulations such as taking square roots of the percentages, the *z*-score transformation had no qualitative impact on the PCA and UPGMA results apart from drawing more attention to five unusual species: *Scapanus townsendii*, which eats substantially more roots and tubers than any other species in the dataset; *Euoticus elegantulus*, which is the only true gumnivore, and three heavily fungivorous sciurids. The clustering results were analysed visually in order to determine the best criteria for establishing quantitative feeding specializations. We performed K-means cluster analysis [27] of the transformed percentages in order to test our classification scheme. Species were grouped into Linnean orders in order to evaluate the degree to which taxonomic attribution is related to diet.

We attempted to test previous feeding classifications based on classic trophic levels. We assigned each feeding resource to the categories of ‘plant’ and ‘animal’. Previous workers using this approach excluded fungi from their datasets [2,3,16,18]. However, we recorded many species that consume a significant amount of fungi. Owing to need for standardization, ‘plants’ included any non-animal food resource and therefore fungi and lichens. ‘Animals’ included both vertebrates and invertebrates.

## 3. Results

**Figure 1.**
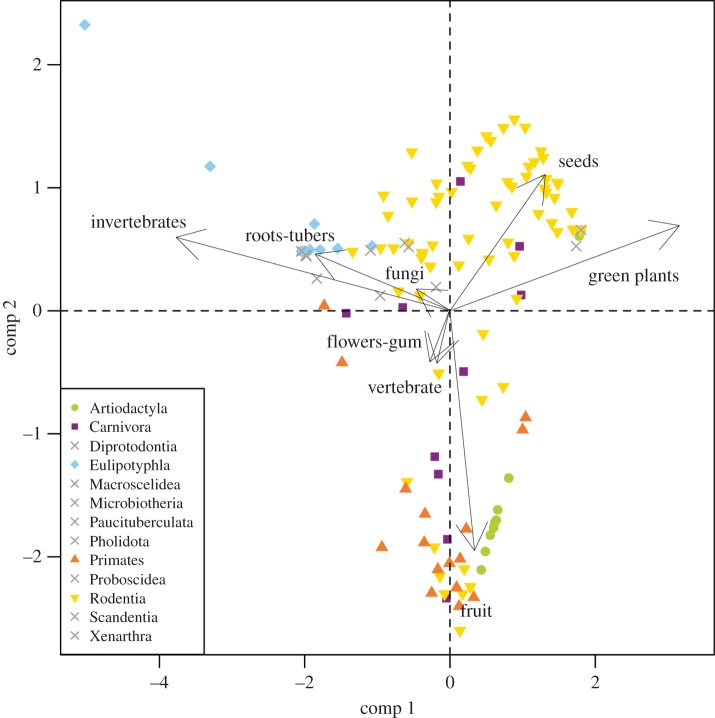
Scores of the two first components of the principal component analysis of dietary data for all the 139 species in the dataset. Raw data are square-root transformed percentages of food items based on stomach content examinations. Arrows indicate the loadings of the two first components of the analysis. Symbols represent taxonomic orders. Crosses indicate orders each represented by less than three species.

The first four components of the PCA together express 65.76% of the variance (electronic supplementary material, S3). The first component (19.4% of the variance) mainly discriminates between animals that feed on invertebrates or roots and tubers and the ones feeding on green plants. The second component (17.53% of the variance) discriminates mainly between fruit-eaters and seed-eaters. Therefore, the two first components alone identify the four major feeding groups (figure 1). The third component (15.19%) discriminates between many of the food resources, with specially high values for fungi and flower-gum eaters. The vertebrate-feeding variable loads strongly only on the fourth component (13.64% of the variance).

**Figure 2.**
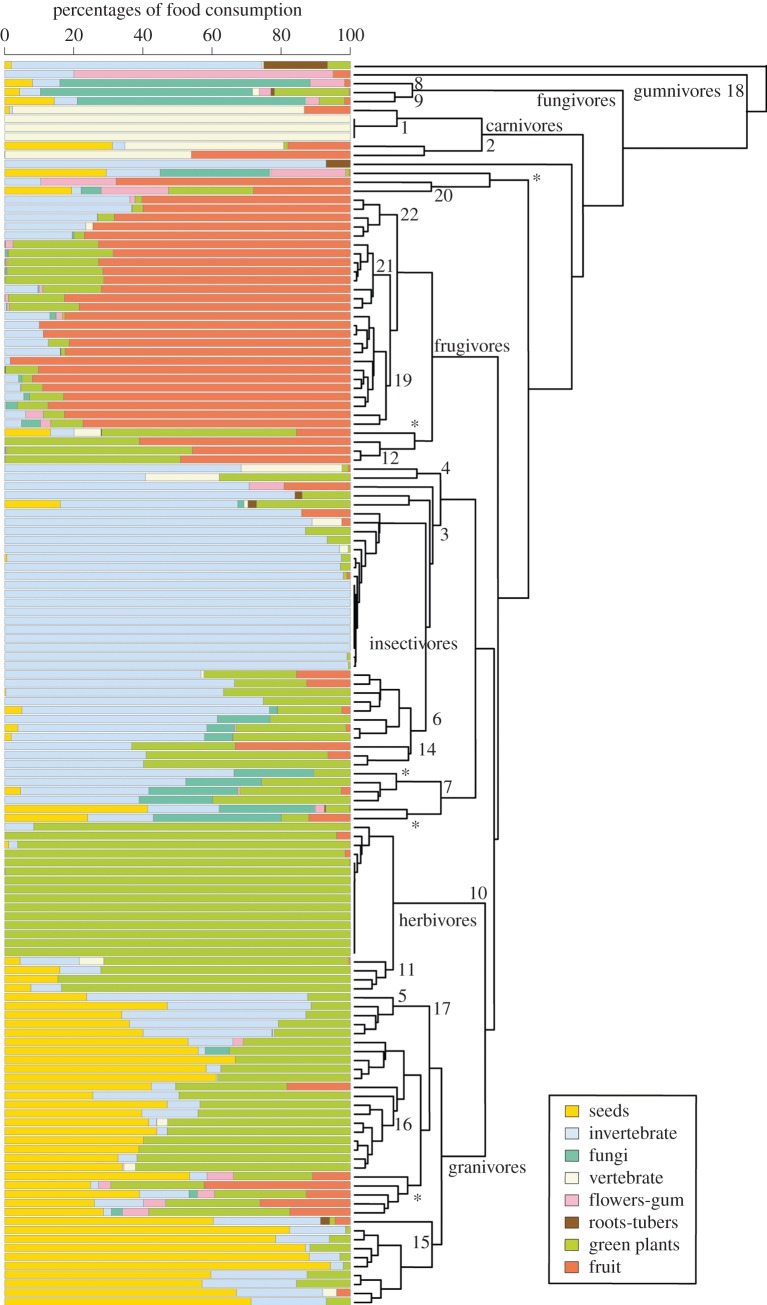
UPGMA cluster analysis based on Euclidean distances of dietary data for the 139 species in the dataset. Raw data are as in figure 1. Numbers represent clusters identified in table 1. Asterisks (*) denote branches containing mixed-feeding species (see text).

The four first components correspond to five major groups of feeding resources: green plants, fruit, seeds, invertebrates and vertebrates (figure 1). It is reasonable to define a species as a pure generalist if none of its feeding resources make up more than 50% of its diet. Just 20 species (14% of the total) have a diet of this kind (figure 2). We might expect a more widespread feeding spectrum or less differentiated feeding groups if most or at least many of the species were truly generalists.

Figure 2 presents a cluster analysis of the 139 species on the dataset. Most of the feeding classifications form consistent and homogeneous clusters. In particular, seven discrete clusters can be differentiated easily. We interpret them as the main dietary specializations: carnivory, insectivory, fungivory, herbivory, granivory, gumnivory and frugivory. The first cluster separates an insectivore species with a high amount of roots and tubers on its diet, the talpid *S. townsendii*. The second cluster identifies the only truly gumnivore species, the primate *E. elegantulus.* The third cluster represents carnivores. Then, a series of minor clusters containing other mammals with peculiar diets separates before the major feeding cluster becomes evident. This is a result of the *z*-score transformation of percentage data, which draws more attention to unusual diet specializations.

Cluster groupings strongly correlate with the results of the PCA, the most readily apparent clusters being the ones formed by carnivores, insectivores, herbivores, granivores and frugivores. Most mammalian species appear to choose between one of these five feeding strategies even if they complement their diet with other resources. The fungi and flowers-gum dietary specializations could be also defined as a feeding category, but they are represented by very few species and present in few environments. Examples include the fungivore chipmunks and *E. elegantulus*.

Special attention must be paid to branches marked with an asterisk (figure 2). Species on these branches are generalists (as defined above). The cluster analysis allocates them based on whatever makes up the plurality of the diet. Similarly, some green plant eaters have been classified within the seed-eater or invertebrate-eater clusters despite having a greater amount of green plants in their diets. Cluster analysis allocates them into these groups because their diets are composed of the same resources, but in different proportions. This results in species consuming relatively low proportions of seeds or invertebrates being plotted into seed- or invertebrate-feeding clusters instead of the green plants cluster.

Table 1 represents the different feeding categories identified in figure 2. The results of the K-means analysis were strongly correlated with the resultant branches of the cluster and with our classification scheme.

**Table 1.** Feeding categories based on the new classification criteria and on the classic trophic level criteria (asterisks indicate those also identified by Eisenberg [11]).

main food resource (>50%)	secondary food resource (20–50%)		cluster in figure 2	classic trophic levels classification
carnivore	—	*	1	carnivore
carnivore	frugivore		2	omnivore
insectivore	—	*	3	carnivore
insectivore	carnivore		4	carnivore
insectivore	granivore		5	omnivore
insectivore	herbivore	*	6	omnivore
insectivore	fungivore		7	omnivore
fungivore	—		8	herbivore
fungivore	herbivore	*	9	herbivore
herbivore	—	*	10	herbivore
herbivore	mixer		11	herbivore
herbivore	frugivore	*	12	herbivore
herbivore	granivore		13	herbivore
herbivore	insectivore		14	omnivore
granivore	—		15	herbivore
granivore	herbivore		16	herbivore
granivore	insectivore		17	carnivore
gumivore	—	*	18	herbivore
frugivore	—		19	herbivore
frugivore	gumnivore	*	20	herbivore
frugivore	herbivore	*	21	herbivore
frugivore	insectivore		22	omnivore
generalists			star	omnivore

The orders Diprotodontia, Macroscelidea, Paucituberculata, Pholidota, Proboscidea, Scadenthia and Xenanthra are each represented by less than three species and hence are all plotted using the same symbol (figure 1). The artiodactyls in our dataset are divided between the fruit and green plant areas in figure 1. Most of them belong to the family Bovidae, which consists almost entirely of herbivore and frugivore species [27]. Other bunodont artiodactyl families that are traditionally described as omnivores (e.g. the Suidae) were not considered in the present analysis, because stomach content data were unavailable. Carnivorans appear to be distributed across most of the feeding spectrum as has already been discussed by Evans *et al*. [2]. Eulipotyphla are all placed in the invertebrate region even though some of them complement their diet with other feeding resources. The talpid *S. townsendii* can be identified at the top-left corner of the chart because of the high amount of roots and tubers in its diet. Primates are distributed between the fruit, invertebrate and green plant resource areas. Rodentia is the most well-represented order in our database with dietary habits covering all major food resource regions. Moreover, 90% of the species in our dataset that fit into our description of generalists belong to the order Rodentia. The highly diverse feeding adaptations of rodents as well as their unspecialized dietary behaviour have been considered to be a primary factor driving their highly successful adaptive radiation [28].

**Figure 3.**
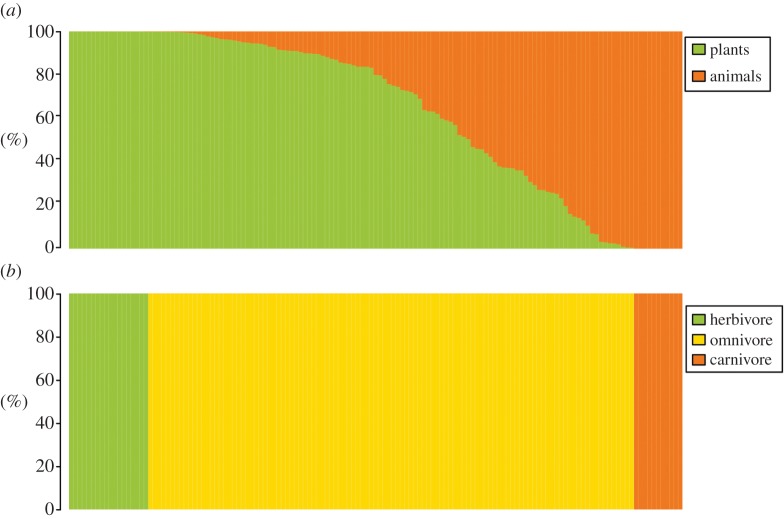
Dendrograms showing (*a*) the proportions of plant and animal resources in each species’ diet and (*b*) feeding classifications based on classic trophic relationships (herbivore–omnivore–carnivore). The 139 species in the dataset are sorted by descending proportions of plant resources in their diets.

In summary, figure 3 shows that classifying the diet of terrestrial mammals within trophic levels would lead to an important loss of information. Very few species have a diet based in a single food category (i.e. are pure herbivores or pure carnivores). Furthermore, establishing simple boundaries between herbivores, omnivores and carnivores would present a challenging task.

## 4. Discussion

### (a) Proposed categories

The main goal of this study is to create a solid platform for research in ecology and palaeoecology concerning dietary adaptations. Ideally, when detailed information is available one would want to perform analyses using food consumption percentages instead of qualitative classifications. In such cases, one might opt for performing multivariate analyses such as those presented here (figures 2 and 3). Although the analyses would be more complex, they would prevent an important loss of information. However, information regarding specific percentages of consumed food resources is simply unavailable or unclear for the vast majority of species. This problem is even more severe when it comes to fossil species, because it is impossible to obtain precise information regarding their dietary habits.

Thus, we really have no choice but to develop a standardized classification scheme for mammalian diets based on what we can observe in real ecosystems. This explains why we have used the major branches of the clustering dendrogram (figure 2) to establish a novel qualitative classification.

To cut to the chase, we propose classifying diets based on the most frequently consumed food resource. We suggest classifying a species as a dietary specialist if a single food resource makes up 50% or more of the diet. Feeding resources consumed with a frequency between 20 and 50% should be also used to categorize the diet. For example, a species consuming 70% green plants and 30% fruits should be classified as an herbivore–frugivore. We classify a species as a ‘generalist’ when none of the feeding resource accounts for at least 50% of the diet.

Based on these simple and highly replicable criteria, we constructed a table showing the different feeding specializations observed in our dataset (table 1). Generalists are distributed throughout the clustering dendrogram (figure 2) depending on the plurality of feeding resources that make up their diets. It might be worth testing whether generalist diets having substantially different compositions correlate with different ecomorphological adaptations.

In this work, herbivore diets have been grouped in a single feeding adaptation, because the main goal is to create a global feeding classification scheme for all terrestrial mammalian faunas. We advocate using the statistically tested classification proposed by Gagnon & Chew [25] when working with highly specialized herbivore mammals or when differentiation between grazers and browsers is necessary for answering research questions.

### (b) Earlier classification schemes

Polis [29] classified the terrestrial vertebrates of a sand community in the Coachella Valley desert in order to assess trophic interactions in a real food web. He proposed a similar approach to ours in that he classified mammalian diets according to primary and secondary feeding categories. However, he considered any food resource that made up over the 10% of the diet to be a ‘primary resource’; while any other food resource was considered to be a ‘secondary resource’ no matter how small the percentage. This classification worked with this particular community because the number of species and the number of interactions were both relatively limited. However, it is hardly applicable in our dataset because there is so much more variation in diets, and a result more feeding resources make up at least 10% of the diet in multiple species.

Some researchers have instead worked with feeding classifications based on classic trophic relationships (herbivores, carnivores and omnivores plus a few variations) [2,3,16,18]. Figure 3 shows the vagueness and oversimplification of this classification criterion. Based on this pattern, it seems self-evident that we should avoid applying the three-way dietary classification whenever possible unless addressing broad trophic level questions. Doing so would not only lead to an overall loss of information but would also specifically fail, because species traditionally described as ‘omnivores’ (table 1) display highly varied diets and are placed here in completely divergent clusters (figure 2).

More realistic classifications should be based on the physical, nutritive and ecological characteristics of food items. Some earlier researchers did distinguish multiple food resources for the purpose of establishing classifications [1,4,11,17]. Among them, Eisenberg [11] presented the most similar classification to ours. He proposed 16 qualitative categories for mammals, some of which can be identified in our results (table 1). The most appreciable difference between his classification and ours involves the degree of resolution: he split up some of our feeding resources (i.e. green plants, invertebrates and vertebrates) into subcategories in order to correlate dietary specializations with substrate utilization. He also overlooked some feeding specializations that we identify in our dataset (i.e. pure granivores or generalists). Finally, Eisenberg [11] described a group that mostly fed on fruits and seeds. This group of species can be identified in figure 2 but they also feed on a considerable amount of green plants, so we have classified them as generalists.

Mendoza *et al*. [17] proposed an intermediate classification scheme for mammalian diets in order to evaluate ecological patterns in the trophic and body size structure of large mammal communities. Carnivores and herbivores were subclassified in more detailed categories that are suitable for studying highly dietary specialized mammalian orders such as carnivorans and artiodactyls. However, they included frugivores, fungivores and gumnivores in a single feeding category and overlooked granivores. Thus, their feeding categories classified in detail some mammalian orders (i.e. carnivorans and artiodactyls), while others were presumed to display no intra-ordinal diet variability (i.e. primates).

Andrews *et al.* [19] also differentiated between insectivores and carnivores and between frugivores and herbivores. In doing so, they also took into account differences in tooth morphology. However, their scheme is only incrementally more detailed than a traditional three-way trophic categorization.

### (c) Frequency of dietary specialization

Our results suggest that terrestrial mammals are in general highly specialized. However, some degree of food mixing may be required for most mammals, because only 23.7% of the records indicate that a single food resource constitutes over 90% of the diet. Singer & Bernays [30] evaluated this topic and suggested that nutritive and non-nutritive factors such as parasite and predator avoidance or ecological restrictions (e.g. competition or food web dynamics) may be responsible for food mixing. Single resource feeders are truly exceptional, and they are usually at the high end of the body mass spectrum. In our dataset only carnivores, insectivores and herbivores display single resource diets, while frugivores, granivores, fungivores and gumnivores seem to require a higher degree of food mixing. Schoener [18] has already noted that the main plant matter in omnivore diets consisted of fruits and seeds. Such diets require less physiological specialization than cellulose-rich herbivore diets.

### (d) Implications for research

Standardized dietary classifications should serve as a cornerstone for research that aims to reconstruct past and present mammalian food webs. Here, we propose an empirically tested scheme that has been erected only after quantitatively analysing dietary data drawn from real ecosystems. That said, our proposal should be considered a work in progress rather than a permanently fixed classification, because the diets of so many species remain to be described in detail. In particular, our criteria do allow adding new feeding groups or even establishing more detailed subcategories when dealing with highly dietary specialized mammalian orders (e.g. artiodactyls or carnivorans). Regardless of the details, however, continuing to work instead with untested dietary classifications would generate unstandardized results and thereby prevent synergy between intellectually overlapping ecological and palaeoecological research programmes.

## Funding statement

The work of S.P-M. was supported by Macquarie University's HDR Project Support Funds. J.A. is the recipient of an Australian Research Council Future Fellowship (project no. FT0992161).

## Supplementary Material

ESM
