# Supplementary material for: Dietary characterization of terrestrial mammals
Source: Proc Biol Sci. 2014 Aug 22;281(1789):20141173. doi: 10.1098/rspb.2014.1173 (PMC4100522; doi:10.1098/rspb.2014.1173)
Supplement: ESM [file rspb20141173supp1.pdf]

ELECTRONIC SUPLEMENTARY MATERIAL 1

Table 1: Summary of stomach content data for the 139 mammalian species analyzed in this study. U=Unspecified.

Order	Family	Species	References	% Seeds	% Invertebrates	% Fungi	% Vertebrates	% Flowers-Gum	% Roots-Tubers	% Green Plants	% Fruit	Sample size
Artiodactyla	Bovidae	Aepyceros melampus	Dunham 1980	0.00	0.00	0.00	0.00	0.00	0.00	100.00	0.00	1737
Artiodactyla	Bovidae	Ammotragus lervia	Gray and Simpson 1980	0.00	0.00	0.00	0.00	0.00	0.00	100.00	0.00	109
Artiodactyla	Bovidae	Antidorcas marsupialis	Davies et al. 1986	0.00	0.00	0.00	0.00	0.00	0.00	98.50	1.50	U
Artiodactyla	Bovidae	Cephalophus callipygus	Gautier-Hion et al. 1980	0.00	0.00	0.10	0.00	0.90	0.00	16.30	82.70	14
Artiodactyla	Bovidae	Cephalophus dorsalis	Gautier-Hion et al. 1980	0.00	0.10	0.10	0.00	0.40	0.00	26.57	72.83	8
Artiodactyla	Bovidae	Cephalophus leucogaster	Gautier-Hion et al. 1980	0.00	0.10	0.10	0.00	2.20	0.00	24.70	72.90	9
Artiodactyla	Bovidae	Cephalophus nigrifrons	Gautier-Hion et al. 1980	0.00	0.10	0.40	0.00	0.20	0.00	27.70	71.60	7
Artiodactyla	Bovidae	Cephalophus silvicultor	Gautier-Hion et al. 1980	0.00	0.10	0.00	0.00	0.00	0.00	28.60	71.30	4
Artiodactyla	Bovidae	Hyemoschus aquaticus	Gautier-Hion et al. 1980	0.00	0.20	0.70	0.00	0.10	0.00	30.37	68.63	19
Artiodactyla	Bovidae	Philantomba monticola	Gautier-Hion et al. 1980	0.00	0.50	0.20	0.00	0.60	0.00	20.28	78.42	16
Artiodactyla	Bovidae	Tragelaphus spekii	Gautier-Hion et al. 1980	0.00	0.00	0.00	0.00	0.00	0.00	99.90	0.10	3
Artiodactyla	Tayassuidae	Tayassu pecari	Sowls 1997	0.00	0.00	0.00	0.00	0.00	0.00	39.00	61.00	34
Carnivora	Canidae	Canis lupus dingo	Newsome et al. 1983	0.00	0.00	0.00	100.00	0.00	0.00	0.00	0.00	230
Carnivora	Canidae	Canis mesomelas	Bothma 1966	0.00	68.40	0.00	29.20	0.00	0.00	1.80	0.60	11
Carnivora	Canidae	Otocyon megalotis	Bothma 1966, Klare et al. 2011	0.00	56.72	0.00	0.90	0.00	0.00	26.82	15.55	185

Carnivora	Canidae	Urocyon cinereoargenteus	Hockman and Chapman 1983	31.11	3.65	0.00	45.93	0.00	0.00	1.25	18.06	63
Carnivora	Canidae	Vulpes lagopus	Anthony et al. 2000	0.00	0.00	0.00	100.00	0.00	0.00	0.00	0.00	100
Carnivora	Canidae	Vulpes vulpes	Hockman and Chapman 1983	1.36	0.94	0.00	84.34	0.00	0.00	0.00	13.36	128
Carnivora	Herpestidae	Helogale hirtula	Hemming 1972	0.00	100.00	0.00	0.00	0.00	0.00	0.00	0.00	5
Carnivora	Mustelidae	Galictis vittata	Bisbal E. 1986	0.00	0.00	0.00	100.00	0.00	0.00	0.00	0.00	U
Carnivora	mustelidae	Martes martes	Ruiz-Olmo 1996	0.00	0.10	0.00	53.90	0.00	0.00	0.00	46.00	42
Carnivora	Mustelidae	Meles meles	Cleary et al. 2011	4.47	17.10	0.00	7.06	0.00	0.00	70.87	0.49	281
Carnivora	Nandinidae	Nandinia binotata	McNab 1995	0.00	10.00	0.00	0.00	0.00	0.00	0.00	90.00	U
Carnivora	Procyonidae	Procyon lotor	Baker et al. 1945	67.00	25.00	0.00	4.00	0.00	0.00	0.00	4.00	23
Carnivora	Ursidae	Ursus arctos	Sato et al. 2005	13.22	6.81	0.00	7.85	0.00	0.18	56.32	15.62	532
Diprotodontia	Burramyidae	Cercartetus caudatus	Flannery and Schouten 1994	0.00	100.00	0.00	0.00	0.00	0.00	0.00	0.00	U
Diprotodontia	Macropodidae	Thylogale stigmatica	Vernes 1995	0.00	0.00	0.00	0.00	0.00	0.00	100.00	0.00	16
Eulipotyphla	Chrysochlorida e	Eremitalpa granti	Fielden et al. 1990, Perrin and Fielden 1999	0.00	93.00	0.00	0.00	0.00	7.00	0.00	0.00	19
Eulipotyphla	Soricidae	Blarina brevicauda	Hahus and Smith 1990	0.65	96.80	0.00	0.00	0.00	0.00	2.55	0.00	
Eulipotyphla	Soricidae	Crocidura cyanea	Monadjem 1997	0.00	100.00	0.00	0.00	0.00	0.00	0.00	0.00	23
Eulipotyphla	Soricidae	Crocidura flavecens	Monadjem 1997	0.00	93.30	0.00	0.00	0.00	0.00	6.70	0.00	5
Eulipotyphla	Soricidae	Crocidura mariquensis	Monadjem 1997	0.00	100.00	0.00	0.00	0.00	0.00	0.00	0.00	U
Eulipotyphla	Soricidae	Myosorex cafer	Monadjem 1997	0.00	74.80	0.00	0.00	0.00	0.00	25.20	0.00	7
Eulipotyphla	Soricidae	Myosorex varius	Monadjem 1997	0.00	97.10	0.00	0.00	0.00	0.00	2.90	0.00	U
Eulipotyphla	Soricidae	Sorex fumeus	Owen 1984	0.00	99.00	0.00	0.00	0.00	0.00	1.00	0.00	4

Eulipotyphla	Soricidae	Sorex hoyi	Long 1974	0.00	100.00	0.00	0.00	0.00	0.00	0.00	0.00	10
Eulipotyphla	Soricidae	Sorex palustris	Beneski and Stinson 1987	0.00	87.00	0.00	0.00	0.00	0.00	13.00	0.00	U
Eulipotyphla	Talpidae	Parascalops breweri	Hallett 1978	0.00	84.00	0.00	0.00	0.00	2.00	14.00	0.00	U
Eulipotyphla	Talpidae	Scapanus townsendii	Carraway et al. 1993	1.91	72.46	0.00	0.59	0.00	18.41	6.64	0.00	100
Eulipotyphla	Talpidae	Talpa europaea	Funmilayo 1979	0.00	100.00	0.00	0.00	0.00	0.00	0.00	0.00	252
Macroscelidea	Macroscelidae	Elephantulus brachyrhynchus	Koontz and Roeper 1983, Leirs et al. 1995	0.00	100.00	0.00	0.00	0.00	0.00	0.00	0.00	U
Macroscelidea	Macroscelidae	Elephantulus rufescens	Hemming 1972	0.30	63.00	0.00	0.00	0.00	0.00	36.70	0.00	23
Macroscelidea	Macroscelidae	Macroscelides proboscideus	Kerley 1992	0.00	98.00	0.00	0.00	0.00	0.00	1.00	1.00	7
Microbiotheria	Microbiotheriidae	Dromiciops gliroides	Meserve et al. 1988	4.96	71.63	2.23	0.00	0.10	0.00	18.64	2.43	71
Paucituberculata	Caenolestidae	Caenolestes fuliginosus	Barkley and Whitaker 1984	0.00	96.90	0.00	2.50	0.00	0.00	0.60	0.00	38
Paucituberculata	Caenolestidae	Rhyncholestes raphanurus	Meserve 1981	3.84	54.60	7.99	0.00	0.51	0.00	31.75	1.31	11
Pholidota	Manidae	Manis crassicaudata	Heath 1995	0.00	100.00	0.00	0.00	0.00	0.00	0.00	0.00	31
Primates	Atelidae	Alouatta seniculus	Guillotin et al. 1994	0.00	0.11	0.12	0.00	0.35	0.00	53.67	45.75	U
Primates	Atelidae	Ateles paniscus	Guillotin et al. 1994	0.00	0.08	0.00	0.08	0.00	0.00	9.60	90.24	60
Primates	Cebidae	Cebus apella	Guillotin et al. 1994	0.00	26.74	0.00	0.19	0.04	0.00	4.75	68.28	44
Primates	Cercopithecidae	Cercocebus agilis	Gautier-Hion et al. 1980	0.00	6.10	0.00	0.00	5.10	0.00	6.10	82.70	97
Primates	Cercopithecidae	Cercopithecus cephus	Gautier-Hion et al. 1980	0.00	12.60	0.00	0.00	0.00	0.00	6.10	81.30	15
Primates	Cercopithecidae	Cercopithecus neglectus	Gautier-Hion et al. 1980	0.00	4.90	5.40	0.00	3.00	0.00	9.40	77.30	62
Primates	Cercopithecidae	Cercopithecus nictitans	Gautier-Hion et al. 1980	0.00	9.60	0.40	0.00	1.00	0.00	17.00	72.00	9
Primates	Cercopithecidae	Cercopithecus pogonias	Gautier-Hion et al. 1980	0.00	16.10	0.10	0.00	0.10	0.00	1.20	82.50	100

Primates	Cercopithecidae	Colobus guereza	Gautier-Hion et al. 1980	0.00	0.10	0.00	0.00	0.00	0.00	50.80	49.10	52
Primates	Cercopithecidae	Mandrillus sphinx	Gautier-Hion et al. 1980	0.00	4.00	1.00	0.00	0.00	0.00	3.00	92.00	5
Primates	Cercopithecidae	Miopithecus talapoin	Gautier-Hion et al. 1980	0.00	36.20	0.00	0.00	1.50	0.00	2.00	60.30	U
Primates	Galagidae	Euoticus elegantulus	Charles-Dominique 1974	0.00	20.00	0.00	0.00	75.00	0.00	0.00	5.00	9
Primates	Galagidae	Galago alleni	Charles-Dominique 1974	0.00	23.47	0.00	2.04	0.00	0.00	0.00	74.49	52
Primates	Galagidae	Galago demidoff	Charles-Dominique 1974	0.00	70.71	0.00	0.00	10.10	0.00	0.00	19.19	12
Primates	Lorisidae	Arctocebus calabarensis	Charles-Dominique 1974	0.00	85.86	0.00	0.00	0.00	0.00	0.00	14.14	55
Primates	Lorisidae	Perodictus potto	Charles-Dominique 1974	0.00	10.42	0.00	0.00	21.88	0.00	0.00	67.71	14
Proboscidea	Elephantidae	Loxodonta africana	Buss 1961	0.00	0.00	0.00	0.00	0.00	0.00	96.00	4.00	41
Rodentia	Heteromyidae	Chaetodipus baileyi	Reichman 1975	88.10	8.90	0.00	0.00	0.00	0.00	3.00	0.00	71
Rodentia	Heteromyidae	Chaetodipus intermedius	Reichman 1975	82.40	16.20	0.00	0.00	0.00	0.00	1.40	0.00	330
Rodentia	Heteromyidae	Dipodomys merriami	Reichman 1975	78.40	15.50	0.00	0.00	0.00	0.00	6.10	0.00	783
Rodentia	Heteromyidae	Perognathus amplus	Reichman 1975	94.30	3.70	0.00	0.00	0.00	0.00	2.00	0.00	1054
Rodentia	Muridae	Abrothrix longipilis	Meserve et al. 1988	16.10	51.27	1.86	1.12	0.00	2.49	27.16	0.00	714
Rodentia	Muridae	Abrothrix sanborni	Meserve et al. 1988	4.62	37.10	25.69	0.00	0.62	0.00	29.39	2.57	5
Rodentia	Muridae	Acomys cahirinus	Hemming 1972	42.44	7.01	0.00	0.00	0.00	0.00	32.23	18.32	55
Rodentia	Muridae	Acomys dimidiatus	Varty 1990	53.00	13.00	0.00	0.00	3.00	0.00	31.00	0.00	17
Rodentia	Muridae	Aethomys crisophilus	Monadjem 1997	58.30	4.20	0.00	0.00	0.00	0.00	37.50	0.00	30
Rodentia	Muridae	Akodon azarae	Ellis et al. 1998	33.92	53.18	0.00	0.00	0.00	0.00	12.90	0.00	10
Rodentia	Muridae	Arvicanthis niloticus	Hemming 1972	0.00	0.10	0.00	0.00	0.00	0.00	99.90	0.00	81

Rodentia	Muridae	Calomys laucha	Ellis et al. 1998	59.62	27.84	0.00	0.00	0.00	0.00	12.54	0.00	22
Rodentia	Muridae	Calomys musculinus	Ellis et al. 1998	57.08	27.32	0.00	0.00	0.00	0.00	15.60	0.00	139
Rodentia	Muridae	Geoxus valdivianus	Meserve et al. 1988	1.91	55.87	8.12	0.00	0.20	0.00	33.90	0.00	141
Rodentia	Muridae	Gerbilliscus leucogaster	Monadjem 1997	25.50	24.90	0.00	0.00	0.00	0.00	49.60	0.00	18
Rodentia	Muridae	Gerbilliscus robustus	Hemming 1972	25.93	14.21	0.00	0.00	6.41	0.00	27.33	26.13	5
Rodentia	Muridae	Gerbillurus paeba	Perrin et al. 1999	0.00	8.41	0.00	0.00	0.00	0.00	91.59	0.00	4
Rodentia	Muridae	Grammomys dolichurus	Varty 1990	56.00	2.00	7.00	0.00	0.00	0.00	35.00	0.00	42
Rodentia	Muridae	Irenomys tarsalis	Meserve et al. 1988	28.50	2.38	3.21	0.00	7.56	0.00	40.83	17.51	8
Rodentia	Muridae	Lemniscomys rosalia	Monadjem 1997	15.40	0.00	0.00	0.00	0.00	0.00	84.60	0.00	10
Rodentia	Muridae	Lorentzimys nouhuysi	Jackson 1992	0.00	38.88	21.28	0.00	0.00	0.00	39.85	0.00	9
Rodentia	Muridae	Loxodontomys micropus	Meserve et al. 1988	19.37	2.75	5.81	0.00	19.47	0.00	24.57	28.03	58
Rodentia	Muridae	Mastomys natalensis	Langham 1983, Monadjem 1997	47.00	9.50	0.00	0.00	0.00	0.00	43.50	0.00	16
Rodentia	Muridae	Maxomys surifer	Monadjem 1997	0.00	40.94	0.00	0.00	0.00	0.00	52.57	6.49	59
Rodentia	Muridae	Micaelamys namaquensis	Langham 1983	40.20	0.00	0.00	0.00	0.00	0.00	59.80	0.00	45
Rodentia	Muridae	Microtus ochrogaster	Hahus and Smith 1990	1.15	2.65	0.00	0.00	0.00	0.00	96.20	0.00	6
Rodentia	Muridae	Mus minutoides	Langham 1983, Kerley 1992	7.61	8.81	0.00	0.00	0.00	0.00	83.58	0.00	57
Rodentia	Muridae	Myotomys unisulcatus	Kerley 1992	0.00	0.00	0.00	0.00	0.00	0.00	100.00	0.00	69
Rodentia	Muridae	Necromys obscurus	Ellis et al. 1998	36.09	43.07	0.00	0.00	0.00	0.00	20.84	0.00	16
Rodentia	Muridae	Oligoryzomys flavescens	Ellis et al. 1998	47.07	41.57	0.00	0.00	0.00	0.00	11.36	0.00	43
Rodentia	Muridae	Oryzomys longicaudatus	Meserve et al. 1988	39.02	14.27	2.44	0.00	5.00	0.00	26.46	12.80	26

Rodentia	Muridae	Otomys angoniensis	Langham 1983	0.00	0.00	0.00	0.00	0.00	0.00	100.00	0.00	37
Rodentia	Muridae	Otomys irroratus	Langham 1983	0.00	0.00	0.00	0.00	0.00	0.00	100.00	0.00	U
Rodentia	Muridae	Peromyscus attwateri	Schmidly 1974	60.36	31.03	0.00	0.00	0.00	2.60	1.60	4.40	5
Rodentia	Muridae	Peromyscus leucopus	Whitaker 1966, Hahus and Smith 1990	40.02	37.31	0.23	0.40	0.00	0.00	22.04	0.00	U
Rodentia	Muridae	Phyllotis darwini	Meserve 1981	41.65	2.38	0.03	3.10	0.00	0.00	52.85	0.00	27
Rodentia	Muridae	Pseudohydromys ellermani	Jackson 1992	0.00	61.50	15.30	0.00	0.00	0.00	23.20	0.00	147
Rodentia	Muridae	Pseudohydromys fuscus	Jackson 1992	0.00	52.40	21.90	0.00	0.00	0.00	25.70	0.00	9
Rodentia	Muridae	Pseudohydromys murinus	Jackson 1992	0.00	66.30	23.10	0.00	0.00	0.00	10.60	0.00	7
Rodentia	Muridae	Pseudohydromys occidentalis	Jackson 1992	0.00	99.50	0.00	0.00	0.00	0.00	0.50	0.00	24
Rodentia	Muridae	Rattus rattus	Langham 1983	61.20	0.40	0.00	0.00	0.00	0.00	38.40	0.00	U
Rodentia	Muridae	Rhabdomys pumilo	Kerley 1992	32.83	5.46	0.00	0.00	0.00	0.00	61.70	0.00	U
Rodentia	Muridae	Taterillus harringtoni	Hemming 1972	24.92	2.30	0.00	0.00	3.45	0.00	26.98	42.34	101
Rodentia	Nesomydae	Dendromus mesomelas	Langham 1983	87.00	1.30	0.00	0.00	0.00	0.00	11.70	0.00	27
Rodentia	Nesomydae	Dendromus mystacalis	Langham 1983	39.70	16.20	0.00	0.00	0.00	0.00	44.10	0.00	5
Rodentia	Nesomydae	Malacothrix typica	Kerley 1992	16.00	11.80	0.00	0.00	0.00	0.00	72.20	0.00	6
Rodentia	Nesomydae	Saccostomus campestris	Langham 1983	66.70	0.00	0.00	0.00	0.00	0.00	33.30	0.00	6
Rodentia	Nesomydae	Saccostomus mearnsi	Varty 1990	44.00	3.00	0.00	0.00	0.00	0.00	53.00	0.00	U
Rodentia	Nesomydae	Steatomys pratensis	Langham 1983	38.80	0.00	0.00	0.00	0.00	0.00	61.20	0.00	U
Rodentia	Octodontidae	Ctenomys mendocinus	Torres-Mura et al. 1989	0.00	0.00	0.00	0.00	0.00	0.00	100.00	0.00	U
Rodentia	Octodontidae	Octodon degus	Meserve et al. 1983	34.07	0.42	0.00	3.39	0.00	0.00	62.11	0.00	U

Rodentia	Octodontidae	Tympanoctomys barrerae	Torres-Mura et al. 1989	0.00	0.00	0.00	0.00	0.00	0.00	100.00	0.00	74
Rodentia	Sciuridae	Callosciurus melanogaster	Whitten 1981	23.71	63.92	0.00	0.00	0.00	0.00	12.37	0.00	U
Rodentia	Sciuridae	Citellus lateralis	Tevis 1953	4.33	6.00	61.33	2.00	3.33	1.00	21.67	0.33	3
Rodentia	Sciuridae	Cynomys ludovicianus	Wydeven and Dahlgren 1982	0.00	0.00	0.00	0.00	0.00	0.00	100.00	0.00	273
Rodentia	Sciuridae	Epixerus ebii	Emmons 1980, Gautier-Hion et al. 1980	0.00	1.60	0.00	0.00	0.00	0.00	0.00	98.40	U
Rodentia	Sciuridae	Funisciurus anerythrus	Emmons 1980	0.00	19.50	0.60	0.00	0.00	0.00	3.00	76.90	8
Rodentia	Sciuridae	Funisciurus isabella	Emmons 1980	0.00	5.53	1.64	0.00	0.00	0.00	9.93	82.91	15
Rodentia	Sciuridae	Funisciurus lemniscatus	Emmons 1980	0.00	36.76	0.10	0.00	0.00	0.00	3.16	59.98	14
Rodentia	Sciuridae	Funisciurus pyrropus	Emmons 1980	0.00	13.10	1.80	0.00	1.80	0.00	0.70	82.60	15
Rodentia	Sciuridae	Heliosciurus rufobrachium	Emmons 1980	0.00	4.53	0.00	0.00	0.20	0.00	6.24	89.03	12
Rodentia	Sciuridae	Lariscus obscurus	Whitten 1981	71.29	21.78	0.00	0.00	0.00	0.00	6.93	0.00	15
Rodentia	Sciuridae	Myosciurus pumilio	Emmons 1980, Gautier-Hion et al. 1980	0.00	36.80	0.00	0.00	0.00	0.00	29.90	33.30	3
Rodentia	Sciuridae	Neotamias townsendi	Tevis 1953	8.00	8.00	72.33	0.00	10.00	0.00	0.00	1.67	6
Rodentia	Sciuridae	Paraxeros poensis	Emmons 1980, Gautier-Hion et al. 1980	0.00	11.15	0.00	0.00	0.00	0.00	0.00	88.85	48
Rodentia	Sciuridae	Protoxerus stangeri	Emmons 1980, Gautier-Hion et al. 1980	0.00	0.31	3.30	0.00	0.00	0.00	8.94	87.44	16
Rodentia	Sciuridae	Sundasciurus lowii	Whitten 1981	0.00	40.21	0.00	0.00	0.00	0.00	59.79	0.00	13
Rodentia	Sciuridae	Tamias amoenus	Tevis 1953	41.33	20.67	27.67	0.17	2.67	0.33	7.00	0.17	7
Rodentia	Sciuridae	Tamias quadrimaculatus	Tevis 1953	14.33	6.67	66.00	0.33	3.67	0.00	7.33	1.67	170
Rodentia	Sciuridae	Tamias speciosus	Tevis 1953	29.33	15.67	31.67	0.33	21.67	0.00	1.00	0.33	165
Rodentia	Sciuridae	Xerus rutilus	Hemming 1972, O'Shea 1991	53.51	5.01	0.00	0.00	7.61	0.00	22.86	11.02	126

Rodentia	Zapodidae	Napaeozapus insignis	Whitaker 1963	24.00	19.00	37.00	0.00	0.00	0.00	8.00	12.00	6
Scandentia	Tupiidae	Tupaia glis	Langham 1983	0.00	66.50	0.00	0.00	0.00	0.00	20.90	12.60	103
Xenarthra	Dasypodidae	Chaetophractus vellerosus	Greegor 1980	0.00	40.68	0.00	21.35	0.00	0.00	37.96	0.00	24
Xenarthra	Dasypodidae	Dasypus novemcinctus	Whitaker 1963	0.00	88.93	0.00	8.58	0.00	0.00	0.00	2.50	84

REFERENCES

Anthony, R. M., N. L. Barten, and P. E. Seiser. 2000. Foods of Arctic foxes *Alopex lagopus* during winter and spring in western Alaska. Journal of Mammalogy 81:820-828.

Baker, R. H., C. C. Newman, and F. Wilke. 1945. Food habits of the raccoon in eastern Texas. Journal of Wildlife Management 9:45-48.

Barkley, L. J. and J. O. Whitaker, Jr. 1984. Confirmation of *Caenolestes* in Peru with Information on Diet. Journal of Mammalogy 65:328-330.

Beneski, J. T., Jr. and D. W. Stinson. 1987. *Sorex palustris*. Mammalian Species 296:1-6.

Bisbal E., F. J. 1986. Food habits of some neotropical carnivores in Venezuela (Mammalia, Carnivora). Mammalia 50:329-340.

Bothma, J. D. P. 1966. Notes on the stomach contents of certain Carnivora (Mammalia) from the Kalahari Gemsbok Park. Koedoe 9:37–39.

Buss, I. O. 1961. Some observations on food habits and behavior of the African Elephant. Journal of Wildlife Management 25:131-148.

Carraway, L. N., L. F. Alexander, and B. J. Verts. 1993. *Scapanus townsendii*. Mammalian Species 434:1-7.

Charles-Dominique, P. 1974 Ecology and feeding behaviour of five sympatric lorisids in Gabon. Pages 131-150 in G. A. D. R. D. Martin, and A. C. Walker, editor. Prosimian Biology. University of Pittsburgh Press, Pittsburgh, PA.

Cleary, G. P., L. A. L. Corner, J. O,ÄôKeeffe, and N. M. Marples. 2011. Diet of the European badger *Meles meles* in the Republic of Ireland: A comparison of results from an analysis of stomach contents and rectal faeces. Mammalian Biology - Zeitschrift für Säugetierkunde 76:470-475.

Davies, R. A. G., P. Botha, and J. D. Skinner. 1986. Diet selected by springbok *Antidorcas marsupialis* and Merino sheep *Ovis aries* during a Karoo drought. Transactions of the Royal Society of South Africa 46:165-176.

Dunham, K. M. 1980. The diet of impala *Aepyceros melampus* in the Sengwa Wildlife Research Area, Rhodesia. Journal of Zoology 192:41-57.

Ellis, B. A., J. N. Mills, G. E. Glass, K. T. McKee, Jr., D. A. Enria, and J. E. Childs. 1998. Dietary habits of the common rodents in an agroecosystem in Argentina. Journal of Mammalogy 79:1203-1220.

Emmons, L. H. 1980. Ecology and resource partitioning among nine species of African rain forest squirrels. Ecological Monographs 50:31-54.

Fielden, L. J., M. R. Perrin, and G. C. Hickman. 1990. Feeding ecology and foraging behaviour of the Namib Desert golden mole, *Eremitalpa granti namibensis* Chrysochloridae. Journal of Zoology 220:367-389.

Flannery, T. F. and P. Schouten. 1994. Possums of the world: a monograph of the Phalangeroidea. GEO Productions in association with the Australian Museum.

Funmilayo, O. 1979. Food consumption preferences and storage in the mole *Talpa europaea*. Acta Theriologica 24:379-390.

Gautier-Hion, A., L. H. Emmons, and G. Dubost. 1980. A comparison of the diets of three major groups of primary consumers of Gabon primates, squirrels and ruminants. Oecologia 45:182-189.

Gray, G. G. and C. D. Simpson. 1980. *Ammotragus lervia*. Mammalian Species 144:1-7.

Greegor, D. H., Jr. 1980. Diet of the little hairy armadillo, *Chaetophractus vellerosus,* of northwestern Argentina. Journal of Mammalogy 61:331-334.

Guillotin, M., G. Dubost, and D. Sabatier. 1994. Food choice and food competition among the three major primate species of French Guiana. Journal of Zoology 233:551-579.

Hahus, S. C. and K. G. Smith. 1990. Food habits of *Blarina*, *Peromyscus*, and *Microtus* in relation to an emergence of periodical cicadas *Magicicada*. Journal of Mammalogy 71:249-252.

Hallett, J. G. 1978. *Parascalops breweri*. Mammalian Species 98:1-4.

Heath, M. E. 1995. *Manis crassicaudata*. Mammalian Species 414:1-4.

Hemming, C. F. 1972. The South Turkana Expedition: scientific papers. VIII. The ecology of South Turkana: a reconnaissance classification. Geographical Journal 138:15-40.

Hockman, J. G. and J. A. Chapman. 1983. Comparative feeding habits of red foxes *Vulpes vulpes* and fray foxes *Urocyon cinereoargenteus* in Maryland. American Midland Naturalist 110:276-285.

Jackson, K. L. a. W., P. A. 1992. The diet of five species of New Guinean rodents. Science in New Guinea 19:77–86.

Kerley, G. I. H. 1992. Trophic status of small mammals in the semi-arid Karoo, South Africa. Journal of Zoology 226:563-572.

Klare, U., J. F. Kamler, and D. W. Macdonald. 2011. The bat-eared fox: a dietary specialist? Mammalian Biology - Zeitschrift für Säugetierkunde 76:646-650.

Koontz, F. W. and N. J. Roeper. 1983. *Elephantulus rufescens*. Mammalian Species 204:1-5.

Langham, N. 1983. Distribution and ecology of small mammals in three rain forest localities of Peninsula Malaysia with particular references to Kedah Peak. Biotropica 15:199-206.

Leirs, H., R. Verhagen, W. Verheyen, and M. R. Perrin. 1995. The biology of *Elephantulus brachyrhynchus* in natural miombo woodland in Tanzania. Mammal Review 25:45-49.

Long, C. A. 1974. *Microsorex hoyi* and *Microsorex thompsoni*. Mammalian Species 33:1-4.

McNab, B. K. 1995. Energy expenditure and conservation in frugivorous and mixed-diet carnivorans. Journal of Mammalogy 76:206-222.

Meserve, P. L. 1981. Trophic Relationships among small mammals in a Chilean semiarid thorn scrub community. Journal of Mammalogy 62:304-314.

Meserve, P. L., B. K. Lang, and B. D. Patterson. 1988. Trophic relationships of small mammals in a Chilean temperate rainforest. Journal of Mammalogy 69:721-730.

Meserve, P. L., R. E. Martin, and J. Rodriguez. 1983. Feeding ecology of two Chilean caviomorphs in a central Mediterranean savanna. Journal of Mammalogy 64:322-325.

Monadjem, A. 1997. Stomach contents of 19 species of small mammals from Swaziland. South African Journal of Zoology 32:23.

Newsome, A., L. Corbett, P. Catling, and R. Burt. 1983. The feeding ecology of the dingo. 1. Stomach contents from trapping in south-eastern Australia, and the non-target wildlife also caught in dingo traps. Wildlife Research 10:477-486.

O'Shea, T. J. 1991. *Xerus rutilus*. Mammalian Species 370:1-5.

Owen, J. G. 1984. *Sorex fumeus*. Mammalian Species 215:1-8.

Perrin, M. R., E. R. Dempster, and C. T. Downs. 1999. *Gerbillurus paeba.* Mammalian Species 606:1-6.

Perrin, M. R., and L. J. Fielden. 1999. *Eremitalpa granti.* Mammalian Species 629:1-4.

Reichman, O. J. 1975. Relation of desert rodent diets to available resources. Journal of Mammalogy 56:731-751.

Ruiz-Olmo, J., López-Martín, J. M. . 1996 Seasonal food of pine marten *Martes martes* L., 1758 in a fir forest of Pyrenean mountains Northeastern Spain In: Mathias, M. L., Santos-Reis, M., Amori, G., Libois, R., Mitchell-Jones, A., Saint-Girons, M.C. Eds.. Proceedings of the I European Congress of Mammalogy. Lisboa , Portugal: 189-196.

Sato, Y., T. Mano, and S. Takatsuki. 2005. Stomach contents of brown bears *Ursus arctos* in Hokkaido, Japan. Wildlife Biology 11: 133-144.

Schmidly, D. J. 1974. *Peromyscus attwateri*. Mammalian Species 48:1-3.

Sowls, L. K. 1997. Javelinas and Other Peccaries: Their Biology, Management, and Use. Texas A&M University Press.

Tevis, L., Jr. 1953. Stomach contents of chipmunks and mantled squirrels in northeastern California. Journal of Mammalogy 34: 316-324.

Torres-Mura, J. C., M. L. Lemus, and L. C. Contreras. 1989. Herbivorous specialization of the South American desert rodent *Tympanoctomys barrerae*. Journal of Mammalogy 70:646-648.

Varty, N. 1990. Ecology of the small mammals in the riverine forests of the Jubba Valley, Southern Somalia. Journal of Tropical Ecology 6:179-189.

Vernes, K. 1995. The diet of the red-legged pademelon *Thylogale stigmatica* Gould (Marsupialia : Macropodidae) in fragmented tropical rainforest, north Queensland, Australia. Mammalia 59: 517-526.

Whitaker, J. O., Jr. 1963. Food, habitat and parasites of the woodland jumping mouse in central New York. Journal of Mammalogy 44: 316-321.

Whitaker, J. O., Jr. 1966. Food of *Mus musculus, Peromyscus maniculatus bairdi* and *Peromyscus leucopus* in Vigo County, Indiana. Journal of Mammalogy 47: 473-486.

Whitten, J. E. J. 1981. Ecological separation of three diurnal squirrels in tropical rainforest on Siberut Island, Indonesia. Journal of Zoology 193: 405-420.

Wydeven, P. R. and R. B. Dahlgren. 1982. A comparison of prairie dog stomach contents and feces using a microhistological technique. Journal of Wildlife Management 46: 1104-1108.

ELECTRONIC SUPLEMENTARY MATERIAL 2

**Feeding categories**	**Feeding resources**
Seeds	Seeds and their tissues
Invertebrates	Arthropods, worms and molluscs
Vertebrates	Vertebrate animals
Fungi	Fungi and lichens
Flowers & Gum	Flowers and gum
Roots & Tubers	Roots and tubers
Green Plants	Angiosperm leaves and branches and ferns
Fruit	Fruits and their tissues

Feeding resources considered in each of the feeding categories

ELECTRONIC SUPLEMENTARY MATERIAL 3

Importance of components:

	**Comp.1**	**Comp.2**	**Comp.3**	**Comp.4**	**Comp.5**	**Comp.6**	**Comp.7**	**Comp.8**
Standard deviation	1.241	1.180	1.098	1.041	0.991	0.945	0.919	0.000
Proportion of Variance	0.194	0.175	0.152	0.136	0.124	0.112	0.106	0.000
Cumulative Proportion	0.194	0.369	0.521	0.658	0.781	0.894	1.000	1.000

Loadings:

**Feeding resources**	**Comp.1**	**Comp.2**	**Comp.3**	**Comp.4**	**Comp.5**	**Comp.6**	**Comp.7**	**Comp.8**
Seeds	0.241	0.436	-0.328	-0.33	0.626			-0.369
Invertebrates	-0.691	0.235	0.174	0.122		0.35	0.148	-0.527
Fungi			-0.611	0.145	-0.438		-0.609	-0.174
Vertebrate		-0.17		-0.828	-0.422	-0.15		-0.267
Flowers and gum		-0.166	-0.604	0.168		-0.217	0.715	-0.11
Roots and tubers	-0.339	0.179	0.163	0.117		-0.888	-0.139	
Green plants	0.579	0.272	0.296	0.324	-0.365		0.129	-0.488
Fruit		-0.767		0.152	0.309		-0.214	-0.483

Importance of the components and loadings of the variables based on a principal component analysis of the stomach content data for the 139 species in this study.
